# P-671. Clinical and Laboratory Findings of Measles in Hospitalized Adolescents and Adults in Romania: A Retrospective Study

**DOI:** 10.1093/ofid/ofae631.867

**Published:** 2025-01-29

**Authors:** Daniela G Neagu, David V Mangaloiu, Violeta Molagic, Catalin Tiliscan, Laurentiu M Stratan, Isabela D Staicu, Aida I Adamescu, Victor Daniel Miron, Oana Sandulescu, Anca Streinu Cercel, Anca C Draganescu, Daniela Pitigoi, Simona Paraschiv, Catalin G Apostolescu, Stefan S Arama, Victoria Arama

**Affiliations:** National Institute of Infectious Diseases „Prof. Dr. Matei Balș”, Bucharest, Romania, Bucuresti, Bucuresti, Romania; 1-Carol Davila University of Medicine and Pharmacy, Bucharest, Romania.2-National Institute of Infectious Diseases „Prof. Dr. Matei Balș”, Bucharest, Romania, Bucuresti, Bucuresti, Romania; 1. Carol Davila University of Medicine and Pharmacy, Bucharest, Romania 2. National Institute of Infectious Diseases „Prof. Dr. Matei Balș”, Bucharest, Romania, Bucuresti, Bucuresti, Romania; 1. Carol Davila University of Medicine and Pharmacy, Bucharest, Romania 2. National Institute of Infectious Diseases „Prof. Dr. Matei Balș”, Bucharest, Romania, Bucuresti, Bucuresti, Romania; 1. Carol Davila University of Medicine and Pharmacy, Bucharest, Romania 2. National Institute of Infectious Diseases „Prof. Dr. Matei Balș”, Bucharest, Romania, Bucuresti, Bucuresti, Romania; National Institute of Infectious Diseases „Prof. Dr. Matei Balș”, Bucharest, Romania., Bucuresti, Bucuresti, Romania; 1. Carol Davila University of Medicine and Pharmacy, Bucharest, Romania 2. National Institute of Infectious Diseases „Prof. Dr. Matei Balș”, Bucharest, Romania, Bucuresti, Bucuresti, Romania; 1. Carol Davila University of Medicine and Pharmacy, Bucharest, Romania 2. National Institute of Infectious Diseases „Prof. Dr. Matei Balș”, Bucharest, Romania, Bucuresti, Bucuresti, Romania; 1. Carol Davila University of Medicine and Pharmacy, Bucharest, Romania 2. National Institute of Infectious Diseases „Prof. Dr. Matei Balș”, Bucharest, Romania., Bucuresti, Bucuresti, Romania; 1. Carol Davila University of Medicine and Pharmacy, Bucharest, Romania 2. National Institute of Infectious Diseases „Prof. Dr. Matei Balș”, Bucharest, Romania, Bucuresti, Bucuresti, Romania; 1. Carol Davila University of Medicine and Pharmacy, Bucharest, Romania 2. National Institute of Infectious Diseases „Prof. Dr. Matei Balș”, Bucharest, Romania, Bucuresti, Bucuresti, Romania; 1. Carol Davila University of Medicine and Pharmacy, Bucharest, Romania 2. National Institute of Infectious Diseases „Prof. Dr. Matei Balș”, Bucharest, Romania., Bucuresti, Bucuresti, Romania; 1. Carol Davila University of Medicine and Pharmacy, Bucharest, Romania 2. National Institute of Infectious Diseases „Prof. Dr. Matei Balș”, Bucharest, Romania, Bucuresti, Bucuresti, Romania; 1. Carol Davila University of Medicine and Pharmacy, Bucharest, Romania 2. National Institute of Infectious Diseases „Prof. Dr. Matei Balș”, Bucharest, Romania, Bucuresti, Bucuresti, Romania; 1. Carol Davila University of Medicine and Pharmacy, Bucharest, Romania 2. National Institute of Infectious Diseases „Prof. Dr. Matei Balș”, Bucharest, Romania, Bucuresti, Bucuresti, Romania; 1. Carol Davila University of Medicine and Pharmacy, Bucharest, Romania 2. National Institute of Infectious Diseases „Prof. Dr. Matei Balș”, Bucharest, Romania, Bucuresti, Bucuresti, Romania

## Abstract

**Background:**

Despite the availability of efficient and safe vaccines, measles remains a significant public health threat worldwide. In Romania, there has been a resurgence of measles since March 2023, leading up to an outbreak in early December, with many cases reported including among the adult population. Consequently, this study aims to present the epidemiological, clinical, and laboratory data of adolescents and adults hospitalized for measles.

**Table 1.**
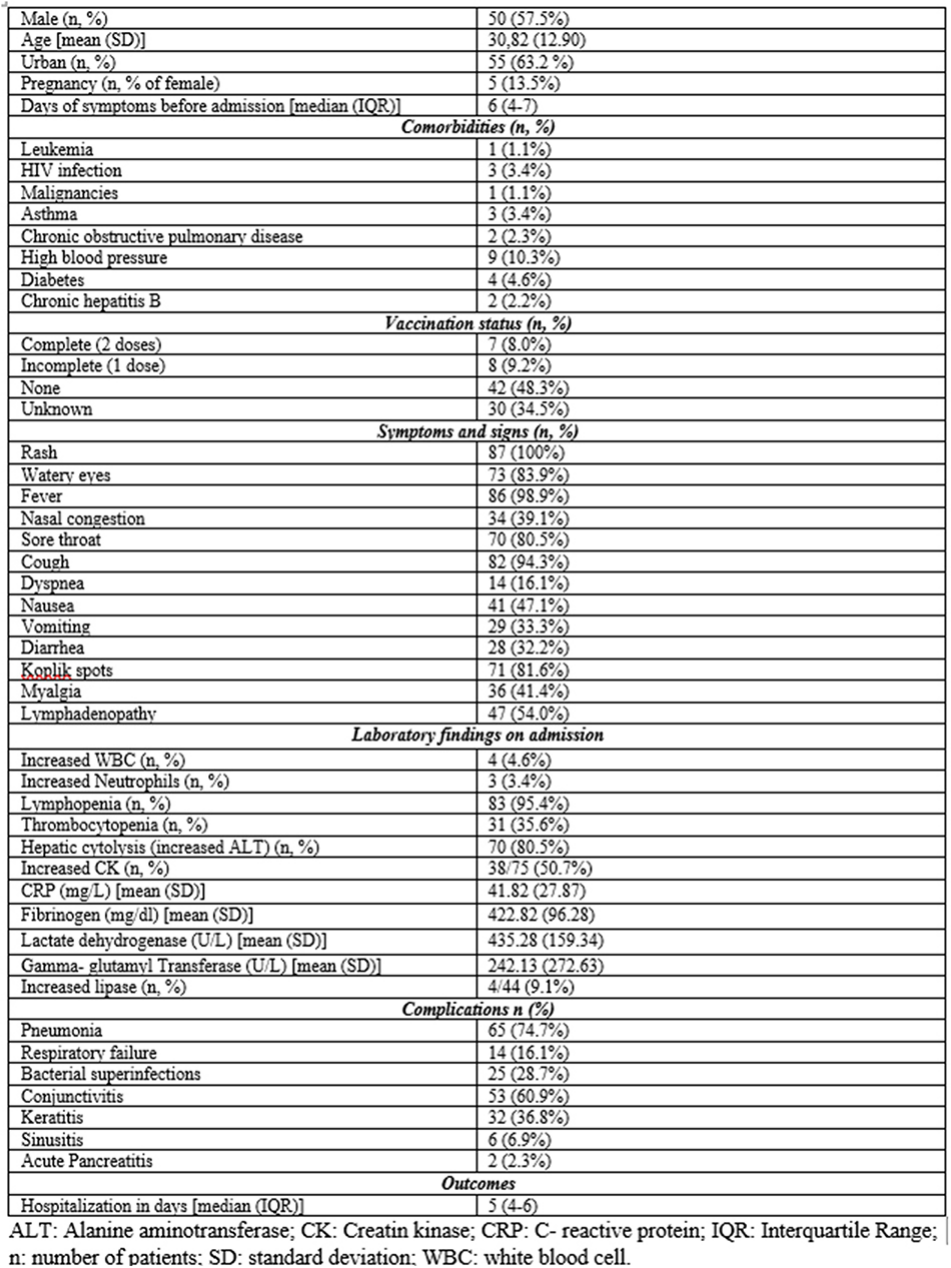
Patient demographics, clinical, paraclinical and complications characteristics.

**Methods:**

We conducted a retrospective analysis which included 87 patients, aged over 14 years, with measles admitted to National Institute of Infectious Disease „Prof. Dr. Matei Balș”, Bucharest, Romania, between July 2023 and April 2024. For all included patients the diagnosis of measles was serologically confirmed. Demographic, epidemiological, clinical and laboratory data were collected for each patient.

**Results:**

Our results are summarized in Table 1. In brief, the mean age of the participants was 30.82 ± 12.90 years and 57.5% were males. Most of them (89.8%) had no chronic conditions. The most frequent symptoms and signs presented at admission were rash (100%), fever (98.9%) and cough (94.3%). Koplik spots were found in 81.6% of patients. A total of 8% of patients had a full history of MMR vaccination, while 9.2% had been vaccinated with only one dose. The most common laboratory parameter findings were increased C-reactive protein (100%), lymphopenia (95.4%) and liver cytolysis (80.5%). The median duration of hospitalization was 5 days. Pneumonia (74.7%) was the most frequent complication and acute respiratory failure (ARF) was reported in 16.1% of patients. The presence of at least one chronic condition increased the risk of ARF 3.5-fold (p=0.036), and prolonged the duration of hospitalization by at least one day.

**Conclusion:**

The ongoing measles outbreak in Romania highlights significant gaps in adolescents and adults vaccination and immunity. The data underline the severe impact of measles in adults, particularly those with unmanaged chronic conditions, leading to extended hospitalization and complications. This study emphasizes the need for improved vaccination strategies and public health policies to mitigate the measles threat effectively.

**Disclosures:**

**All Authors**: No reported disclosures

